# CircRNA circ‐IQGAP1 Knockdown Alleviates Interleukin‐1β‐Induced Osteoarthritis Progression *via* Targeting miR‐671‐5p/TCF4

**DOI:** 10.1111/os.12923

**Published:** 2021-03-05

**Authors:** Peng Xi, Cai‐lin Zhang, Shi‐yan Wu, Lei Liu, Wen‐ju Li, Yi‐mei Li

**Affiliations:** ^1^ Pain Department the First Affiliated Hospital ofXinjiang Medical University Urumqi China

**Keywords:** circ‐IQGAP1, miR‐671‐5p, Osteoarthritis, TCF4

## Abstract

**Objective:**

To explore the function of circular RNA IQ motif‐containing GTPase‐activating protein 1 (circ‐IQGAP1) in interleukin (IL)‐1β‐induced osteoarthritis (OA) model and to explore whether circ‐IQGAP1 can modulate microRNA‐671‐5p (miR‐671‐5p) and transcription factor 4 (TCF4) to regulate chondrocyte apoptosis, inflammatory injury, and extracellular matrix degradation.

**Methods:**

The cartilage tissues were collected from 32 OA patients or normal subjects. Human chondrocyte CHON‐001 cells were challenged *via* different doses of IL‐1β for 24 hours. CHON‐001 cells were transfected with circ‐IQGAP1 overexpression vector, TCF4 overexpression vector, small interfering RNA (siRNA) for circ‐IQGAP1, miR‐671‐5p mimic, miR‐671‐5p inhibitor or corresponding negative controls. Circ‐IQGAP1, miR‐671‐5p and TCF4 abundances in cartilage tissues or CHON‐001 cells were examined *via* quantitative reverse transcription polymerase chain reaction (qRT‐PCR) or western blot. Cell viability was investigated by 3‐(4, 5‐dimethylthiazol‐2‐yl)‐2, 5‐diphenyltetrazolium bromide (MTT). Cell apoptosis was measured by flow cytometry. The inflammatory injury was analyzed by the secretion levels of inflammatory cytokines (IL‐6, IL‐8 and tumor necrosis factor‐α [TNF‐α]) by enzyme‐linked immunosorbent assay (ELISA). The extracellular matrix degradation was evaluated by expression of aggrecan and matrix metalloproteinase 13 (MMP13) *via* western blot. The target relationship of miR‐671‐5p and circ‐IQGAP1 or TCF4 was analyzed *via* dual‐luciferase reporter and RNA immunoprecipitation (RIP) analyses.

**Results:**

Circ‐IQGAP1 abundance was enhanced in the cartilage tissues from OA patients compared with normal subjects (*n* = 32), and its expression was increased in CHON‐001 cells after treatment of IL‐1β in a dose‐dependent pattern. MiR‐671‐5p expression was decreased in the cartilage tissues from OA patients (*n* = 32) and IL‐1β‐challenged CHON‐001 cells. MiR‐671‐5p expression was negatively associated with circ‐IQGAP1 level in OA patients. Circ‐IQGAP1 silence mitigated IL‐1β‐caused chondrocyte viability reduction, apoptosis promotion, secretion of inflammatory cytokine (IL‐6, IL‐8 and TNF‐α), and extracellular matrix degradation (reduction of aggrecan and increase of MMP13). MiR‐671‐5p was targeted and inhibited *via* circ‐IQGAP1. MiR‐671‐5p knockdown attenuated the influence of circ‐IQGAP1 interference on IL‐1β‐caused chondrocyte apoptosis, inflammatory injury, and extracellular matrix degradation. TCF4 was targeted *via* miR‐671‐5p, and TCF4 expression was increased in the cartilage tissues from OA patients (*n* = 32) and IL‐1β‐challenged CHON‐001 cells. TCF4 abundance in OA patients was negatively correlated with miR‐671‐5p expression. MiR‐671‐5p overexpression alleviated IL‐1β‐mediated chondrocyte apoptosis, inflammatory injury, and extracellular matrix degradation *via* decreasing TCF4 expression. Circ‐IQGAP1 silence reduced TCF4 expression *via* regulating miR‐671‐5p in IL‐1β‐challenged CHON‐001 cells.

**Conclusion:**

Circ‐IQGAP1 knockdown attenuated IL‐1β‐caused chondrocyte apoptosis, inflammatory injury, and extracellular matrix degradation. Circ‐IQGAP1 could regulate miR‐671‐5p/TCF4 axis to modulate IL‐1β‐caused chondrocyte damage. Circ‐IQGAP1 might act as a new target for the treatment of OA.

## Graphical abstract

Circ‐IQGAP1 promotes IL‐1β‐induced chondrocyte apoptosis, inflammation, and extracellular matrix degradation by miR‐671‐5p/TCF4 axis in osteoarthritis.

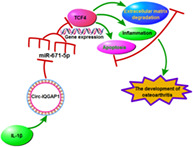

## Introduction

Osteoarthritis (OA) is a prevalent painful disorder characterized by cartilage degradation in the elderly population, and this disease is difficult to treat worldwide^1^. With the effect of aging and obesity, this syndrome has become more prevalent in recent years, and about 250 mn people are affected worldwide^2^. Pain (initiated from free axonal endings of synovium, periosteum bone, and tendons) and disability are the major symptoms of OA, which can induce huge health burdens in patients^3^, ^4^. The chondrocytes are the resident cells for maintaining the extracellular matrix in articular cartilage, and the damage of chondrocytes is correlated with OA progression, including chondrocyte apoptosis, inflammatory injury, and extracellular matrix destruction^5^, ^6^, ^7^, ^8^. Apoptosis is an active process of cell death in development, and chondrocyte apoptosis may lead to the failure of articular cartilage in OA^6^. Moreover, the inflammatory processes by secretion of multiple inflammatory cytokines can promote OA progression *via* chondrocyte dysregulation^7^. Additionally, the extracellular matrix degradation contributes to the destruction of articular cartilage, which is also a key factor for OA development^8^. Interleukin‐1β (IL‐1β) is a major cytokine that is present in the joint following the inflammation and is involved in the dysfunction of chondrocytes in OA, and it is widely used for the induction of OA‐like processes and the establishment of OA model *in vitro* ^9^, ^10^. Hence, exploring the mechanism of IL‐1β‐caused dysfunction of chondrocytes might help to understand the pathogenesis of OA, and find new targets for the treatment of OA.

The noncoding ribonucleic acids (RNAs) that do not encode proteins are important players in the regulation of gene expression, and are relevant to the development of multiple diseases, including chondrocyte function in OA^11^. Circular RNAs (circRNAs) are a type of closed noncoding RNA molecule without a 3′ and 5′ end that can sponge microRNAs to derepress mRNAs in eukaryotic cells, which play key roles in the development of OA^12^. CircRNAs can serve as important biomarkers for the diagnosis of OA^13^. Moreover, lots of circRNAs are abnormally expressed in the cartilage of OA patients, and they have potential roles in regulating cartilage degradation^14^. For example, circRNA serpin family E member 2 (circSERPINE2) could interact with microRNA‐1271 (miR‐1271) and E26 transformation‐specific‐related gene (ERG), and attenuate OA progression by decreasing chondrocyte apoptosis and extracellular matrix degradation^15^. Furthermore, circRNA.33186 could promote chondrocyte apoptosis and extracellular matrix degradation and contribute to OA progression in mice *via* sponging microRNA‐127‐5p (miR‐127‐5p) and matrix metalloproteinase 13 (MMP13)^16^. In addition, circRNA transmembrane BAX inhibitor motif containing 6 (circ‐TMBIM6) could promote chondrocyte extracellular matrix degradation in OA *via* regulating microRNA‐27a (miR‐27a) and MMP13^17^. Additionally, a microarray analysis shows that circRNA IQ motif‐containing GTPase‐activating protein 1 (circ‐IQGAP1) is a dysregulated circRNA in OA, and it is reported that this circRNA has important clinical significance^18^. Nevertheless, how and whether circ‐IQGAP1 regulates OA progression is largely uncertain.

MicroRNAs (miRNAs) are short (~22 nucleotides in length) RNA molecules that regulate mRNA degradation and translation repression, which have important roles in regulating cartilage function and OA progression^19^. Moreover, many miRNAs are abnormally expressed in OA patients and play key roles in OA development, such as miR‐138‐5p, miR‐146a‐5p, miR‐335‐5p, and miR‐9‐5p^20^. It has been reported that miR‐671‐5p, a downregulated miRNA in OA patients, could weaken chondrocyte apoptosis and extracellular matrix degradation in chondrocytes and mitigate OA progression in a murine model^21^. Nevertheless, the mechanism of miR‐671‐5p is complex, and whether it is involved in circ‐IQGAP1‐addressed mechanism in regulating chondrocyte damage is unclear.

Transcription factor 4 (TCF4) on human chromosome 18 is a common risk gene for various diseases in humans^22^. A previous study suggested that TCF4 was associated with the development of knee OA and might serve as a potential target for the treatment of OA^23^. Moreover, as a target of miR‐93‐5p, TCF4 could promote IL‐1β‐caused chondrocyte apoptosis and extracellular matrix degradation in OA^24^. In addition, TCF4 could promote the inflammatory response and apoptosis in chondrocytes *via* regulating the AMPK/NF‐κB pathway^25^. Furthermore, TCF4 might contribute to cartilage destruction *via* increasing osteopontin (OPN)^26^. However, whether TCF4 is required for circ‐IQGAP1 in the regulation of OA development is unknown.

The bioinformatics analysis was processed based on StarBase software online (http://starbase.sysu.edu.cn/), which predicted that miR‐671‐5p might bind to both circ‐IQGAP1 and TCF4 because of the presence of complementary sites. Thus, we hypothesized that circ‐IQGAP1 might target TCF4 by competitively binding tomiR‐671‐5p, regulating IL‐1β‐caused damage in chondrocytes. In this research, we established an *in vitro* model of OA using IL‐1β‐challenged chondrocytes (CHON‐001) as previously reported^27^, ^28^, ^29^. The purpose of this study was: (i) to investigate the function of circ‐IQGAP1 on IL‐1β‐caused chondrocyte apoptosis, inflammatory injury, and extracellular matrix degradation; (ii) to analyze the regulatory network of circ‐IQGAP1/miR‐671‐5p/TCF4 in chondrocytes; and (iii) to explore whether circ‐IQGAP1 regulated the miR‐671‐5p/TCF4 axis to modulate chondrocyte apoptosis, inflammatory injury, and extracellular matrix degradation. This study might provide new insight into the pathology of chondrocyte damage in OA.

## Methods

### Cartilage Tissue Collection

Thirty‐two cartilage tissues were collected from knee OA patients in the First Affiliated Hospital of Xinjiang Medical University. The normal cartilage tissues (*n* = 32) were obtained during total hip replacement from age‐matched patients with femoral neck fractures who have no symptom of OA. All tissues were verified by histopathological examination. The OA patients were diagnosed in line with the American College of Rheumatology. The surgeries were performed *via* the same team of orthopaedists. The cartilage tissues were used for RNA isolation. All subjects provided written informed consent. This research was permitted *via* the ethics committee of the First Affiliated Hospital of Xinjiang Medical University following the guidelines of the Helsinki Declaration.

### Cell Culture and Exposure to IL‐1β

Human chondrocyte CHON‐001 cells were provided *via* BeNa Culture Collection (Beijing, China) and grew in DMEM (Procell, Wuhan, China) with 10% fetal bovine serum (Thermo Fisher, Waltham, MA, USA) and 0.1 mg/mL G‐418 (Solarbio, Beijing, China) at 37 °C and 5% CO_2_.

To mimic OA‐like injury, CHON‐001 cells were challenged *via* various doses (0, 1, 5, or 10 ng/mL) of IL‐1β (Amyjet, Wuhan, China) for 24 h.

### Quantitative Reverse Transcription Polymerase Chain Reaction (qRT‐PCR)

Total RNA was isolated through Trizol reagent (Applygen, Beijing, China) in accordance with the procedures in a previous study^30^. The cDNA was synthesized using the specific reverse transcription kit (Takara, Tokyo, Japan), and then mixed with SYBR (Solarbio) and specific primers (Genscript, Nanjing, China) for qRT‐PCR. The primer sequences were shown in Table 1. U6 or GAPDH served as a control. The relative RNA expression was analyzed *via* 2^‐ΔΔCt^ method^31^.

**TABLE 1:** The primer sequences for qRT‐PCR in this study

Name	Sequence (5′‐3′)	
	Forward	Reverse
miR‐671‐5p	AGGAAGCCCTGGAGGG	GAACATGTCTGCGTATCTC
U6	TCGCTTCGGCAGCACATATAC	TATGGAACGCTTCACGAATTTG
circ‐IQGAP1	GGGCATCTTGGCTAATGAAC	CCGAGTCTCTGCTGAGGAAG
TCF4	CAAAAACCAGAGCCAGGTGC	GGAGCATAGACTGAAGATGGCA
GAPDH	GACAGTCAGCCGCATCTTCT	GCGCCCAATACGACCAAATC

### Cell Transfection

The circ‐IQGAP1 overexpression vector was constructed *via* inserting circ‐IQGAP1 sequence into pCD5‐ciR vector (Geneseed, Guangzhou, China), and the pCD5‐ciR vector was used as negative control (vector). The TCF4 overexpression vector was generated *via* cloning TCF4 sequence into pcDNA3.1 vector (Invitrogen, Carlsbad, CA, USA), and the pcDNA3.1 alone served as negative control (pcDNA). The siRNA for circ‐IQGAP1 (si‐circ‐IQGAP1, 5′‐AUGAAGCCGCAUGGACCCCGG‐3′), negative control of siRNA (si‐NC) (5′‐UUCUCCGAACGUGUCACGUTT‐3′), miR‐671‐5p mimic (5′‐AGGAAGCCCUGGAGGGGCUGGAG‐3′), negative control of mimic(miR‐NC, 5′‐CGAUCGCAUCAGCAUCGAUUGC‐3′), miR‐671‐5p inhibitor(anti‐miR‐671‐5p, 5′‐CUCCAGCCCCUCCAGGGCUUCCU‐3′), and negative control of inhibitor (anti‐miR‐NC, 5′‐CUAACGCAUGCACAGUCGUACG‐3′) were provided *via* Biosyntech (Suzhou, China). CHON‐001 cells were transfected with the constructed vectors or oligonucleotides and were collected for further experiments after 24 h post‐transfection.

### 3‐(4, 5‐dimethylthiazol‐2‐yl)‐2, 5‐diphenyltetrazolium Bromide (MTT)

Cell viability was tested *via* MTT analysis. After the indicated transfection or treatment, 1 × 10^4^ CHON‐001 cells were added into 96‐well plates, and then incubated for 24, 48, or 72 h, followed by incubating with 0.5 mg/mL MTT (Beyotime, Shanghai, China) for 4 h. Next, the optical density (OD) value was examined *via* a microplate reader (Molecular Devices, San Jose, CA, USA) with a wavelength of 570 nm.

### Flow Cytometry

Cell apoptosis was examined with an Annexin V‐FITC apoptosis kit (Thermo Fisher). After the indicated transfection or treatment, 2 ×10^5^ CHON‐001 cells were added in six‐well plates and incubated for 72 h. Next, cells were collected and interacted with Annexin V‐FITC binding buffer, followed by dying with Annexin V‐FITC and propidium iodide. The apoptotic cells (Annexin V‐FITC^+^/PI^−/+^) were detected *via* a flow cytometer (Agilent, Hangzhou, China). The apoptotic rate was expressed as a percentage of apoptotic cells.

### Enzyme Linked Immunosorbent Assay (ELISA)

The secretion of inflammatory cytokines was detected *via* ELISA. After the indicated transfection or treatment, 5 × 10^4^ CHON‐001 cells were added in 24‐well plates and cultured for 72 h. Next, the medium was collected and applied to the detection of IL‐6, IL‐8, and tumor necrosis factor‐α (TNF‐α) using the specific ELISA kit (R&D Systems, Minneapolis, MN, USA) following the instruction of manufacturer. The concentration of these cytokines was detected *via* a microplate reader.

### Western Blot

Protein was isolated using RIPA buffer (Solarbio), and quantified with a BCA kit (Beyotime, Shanghai, China). The protein samples were separated *via* SDS‐PAGE and transferred to nitrocellulose membranes (Bio‐Rad, Hercules, CA, USA). The membranes were blocked using 5% fat‐free milk, and then interacted with antibody for aggrecan (ab36861, 1:2000 dilution, Abcam, Cambridge, MA, USA), MMP13 (ab39012, 1:3000 dilution, Abcam), TCF4 (ab130014, 1:300 dilution, Abcam) overnight, and IgG conjugated *via* HRP (ab6721, 1:10000 dilution, Abcam) for 2 h. The GAPDH (ab9485, 1:5000 dilution, Abcam) was employed as a loading reference. The blots were exposed to ECL reagent (Abcam), and then tested *via* Quantity One software (Bio‐Rad).

### Dual‐luciferase Reporter Analysis and RNA Immunoprecipitation (RIP)

The targets of circ‐IQGAP1 and miR‐671‐5p were predicted using StarBase (http://starbase.sysu.edu.cn/)^32^. The target correlation of miR‐671‐5p and circ‐IQGAP1 or TCF4 was analyzed *via* the dual‐luciferase reporter and RIP analyses. The wild‐type sequence of circ‐IQGAP1 or TCF4 containing miR‐671‐5p complementary sites was inserted into the pMIR‐REPORT vectors (Thermo Fisher), generating the luciferase reporter vectors WT‐circ‐IQGAP1 or TCF4 3′UTR‐WT. The mutant‐type luciferase reporter vectors MUT‐circ‐IQGAP1 or TCF4 3′UTR‐MUT were obtained *via* mutating the seed sites. These constructs, control vectors, and miR‐671‐5p mimic or miR‐NC were transfected into CHON‐001 cells for 24 h. The luciferase activity was examined with a dual‐luciferase analysis kit (Thermo Fisher).

RIP analysis was conducted with a Magna RIP kit (Millipore, Billerica, MA, USA). 1 × 10^7^ CHON‐001 cells were lysed and incubated with magnetic beads conjugated *via* Ago2 antibody. IgG served as a negative control. The enrichment abundances of circ‐IQGAP1, miR‐671‐5p, and TCF4 were measured *via* qRT‐PCR.

### Statistical Analysis

The experiments were carried out three times, with three replicates of each reaction, unless otherwise indicated. The data were presented as mean ± SD. Statistical analysis was performed *via* GraphPad Prism 6 (GraphPad Inc., La Jolla, CA, USA). The difference was analyzed *via* Student's *t*‐test or ANOVA with Tukey test as appropriate. *P* < 0.05 indicated the significant difference.

## Results

### circ‐IQGAP1Expression is Increased and miR‐671‐5p Abundance is Declined in OA Patients and IL‐1β‐Challenged CHON‐001 Cells

**Fig. 1:**
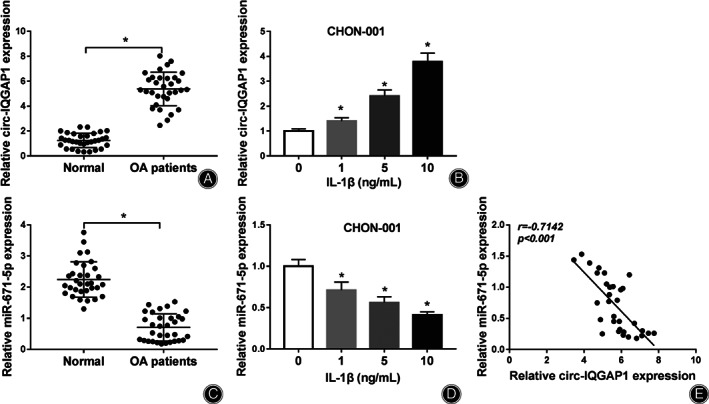
circ‐IQGAP1 and miR‐671‐5p expression in OA. (A) circ‐IQGAP1 abundance in the cartilage tissues of OA or normal patients. *n* = 32. (B) circ‐IQGAP1 level in CHON‐001 cells after treatment of various doses of IL‐1β for 24 h. (C) miR‐671‐5p abundance in the cartilage tissues of OA or normal patients. *n* = 32. (D) miR‐671‐5p expression in CHON‐001 cells after treatment of various doses of IL‐1β for 24 h. (E) The linear correlation of circ‐IQGAP1 and miR‐671‐5p in OA patients. ^*^ *P* < 0.05.

To test whether circ‐IQGAP1 and miR‐671‐5p were implicated in OA progression, their levels were detected in OA patients and IL‐1β‐induced OA model. As shown in Fig. 1A, circ‐IQGAP1 expression was evidently elevated in the cartilage samples of OA patients in comparison to normal subjects. Moreover, circ‐IQGAP1 abundance in CHON‐001 cells was progressively elevated *via* treatment of IL‐1β in a dose‐dependent manner (Fig. 1B). Additionally, miR‐671‐5p level was markedly reduced in OA patients in comparison to normal subjects (Fig. 1C). Furthermore, miR‐671‐5p abundance was significantly declined after exposure to IL‐1β (Fig. 1D). Besides, miR‐671‐5p level in OA patients was negatively correlated with circ‐IQGAP1 (*r* = −0.7142, *P* < 0.001) (Fig. 1E). These results suggested that the dysregulated circ‐IQGAP1 and miR‐671‐5p might be involved in OA progression.

### circ‐IQGAP1 Knockdown Weakens IL‐1β‐Caused Cell Apoptosis, Inflammatory Injury, and Extracellular Matrix Degradation

**Fig. 2:**
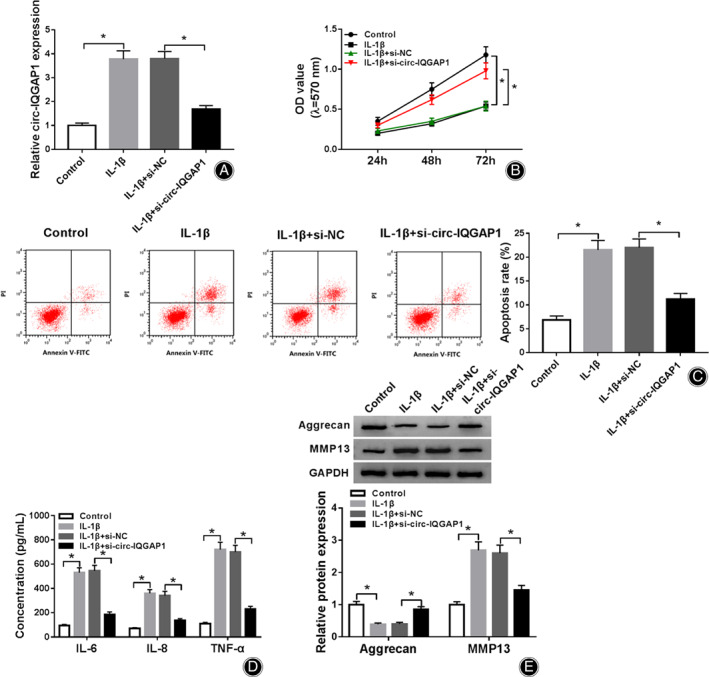
The influence of circ‐IQGAP1 on IL‐1β‐caused cell apoptosis, inflammatory injury, and extracellular matrix degradation. circ‐IQGAP1 expression (A), cell viability (B), apoptosis (C), IL‐6, IL‐8 and TNF‐α levels (D) and aggrecan and MMP13 abundances (E) were detected in CHON‐001 cells with transfection of si‐NC or si‐circ‐IQGAP1 after treatment of IL‐1β. ^*^ *P* < 0.05.

To analyze the function of circ‐IQGAP1 in IL‐1β‐caused chondrocyte injury, CHON‐001 cells were transfected with si‐NC or si‐circ‐IQGAP1 prior to treatment of 10 ng/mL of IL‐1β. As displayed in Fig. 2A, the transfection of si‐circ‐IQGAP1 effectively reduced circ‐IQGAP1 abundance. Furthermore, circ‐IQGAP1 knockdown attenuated IL‐1β‐caused viability reduction (Fig. 2B). Additionally, circ‐IQGAP1 down‐regulation weakened IL‐1β‐caused apoptosis (Fig. 2C). Moreover, circ‐IQGAP1 silence mitigated IL‐1β‐triggered secretion of IL‐6, IL‐8, and TNF‐α (Fig. 2D). Besides, circ‐IQGAP1 interference alleviated IL‐1β‐caused extracellular matrix degradation *via* increasing aggrecan and decreasing MMP13 (Fig. 2E). These data indicated that circ‐IQGAP1 silence attenuated IL‐1β‐induced chondrocyte injury.

### miR‐671‐5p is Targeted *via* circ‐IQGAP1

**Fig. 3:**
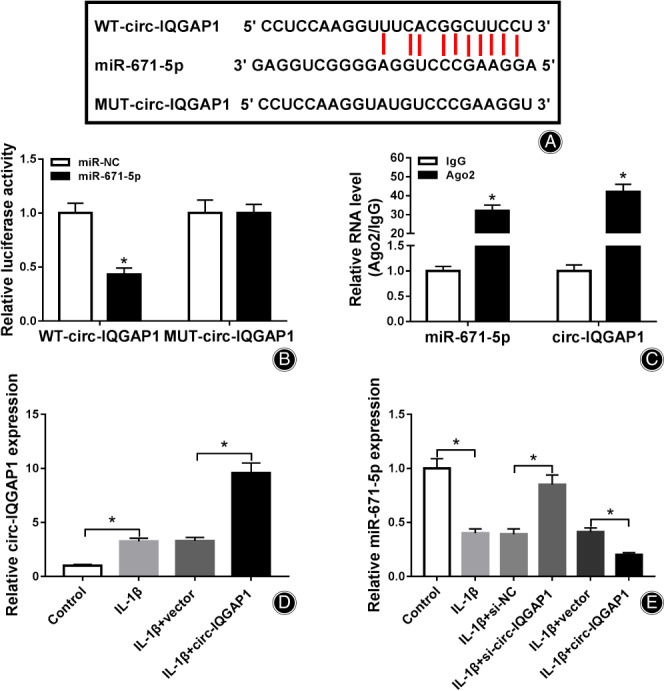
The target correlation of circ‐IQGAP1 and miR‐671‐5p. (A) The target sequence of circ‐IQGAP1 and miR‐671‐5p. (B) Luciferase activity was detected in CHON‐001 cells with transfection of WT‐circ‐IQGAP1 or MUT‐circ‐IQGAP1 and miR‐671‐5p mimic or miR‐NC. (C) circ‐IQGAP1 and miR‐671‐5p abundances after RIP. (D) circ‐IQGAP1 expression in CHON‐001 cells with transfection of vector or circ‐IQGAP1 overexpression vector after treatment of IL‐1β. (E) miR‐671‐5p abundance in CHON‐001 cells with transfection of si‐NC, si‐circ‐IQGAP1, vector or circ‐IQGAP1 overexpression vector after treatment of IL‐1β. ^*^ *P* < 0.05.

To explore the potential regulatory network mediated *via* circ‐IQGAP1, the targets of circ‐IQGAP1 were analyzed *via* StarBase. The target sequence of circ‐IQGAP1 and miR‐671‐5p was shown in Fig. 3A. To validate this network, we constructed the luciferase reporter vectors WT‐circ‐IQGAP1 and MUT‐circ‐IQGAP1 and detected the luciferase activity. Results showed that miR‐671‐5p overexpression caused about 60% loss of luciferase activity of WT‐circ‐IQGAP1, but it did not affect the luciferase activity of MUT‐circ‐IQGAP1 (Fig. 3B). Furthermore, the RIP analysis exhibited that circ‐IQGAP1 and miR‐671‐5p could be enriched in the same complex *via* Ago2 RIP (Fig. 3C). Besides, the influence of circ‐IQGAP1 on miR‐671‐5p expression was assessed in CHON‐001 cells treated *via* IL‐1β. The efficacy of circ‐IQGAP1 overexpression vector was identified in Fig. 3D. miR‐671‐5p abundance was evidently up‐regulated *via* circ‐IQGAP1 knockdown and declined *via* circ‐IQGAP1 overexpression (Fig. 3E). These results suggested that circ‐IQGAP1 could target miR‐671‐5p.

### miR‐671‐5p Knockdown Reverses the Influence of circ‐IQGAP1 Silence on IL‐1β‐Caused Cell Apoptosis, Inflammatory Injury, and Extracellular Matrix Degradation

**Fig. 4:**
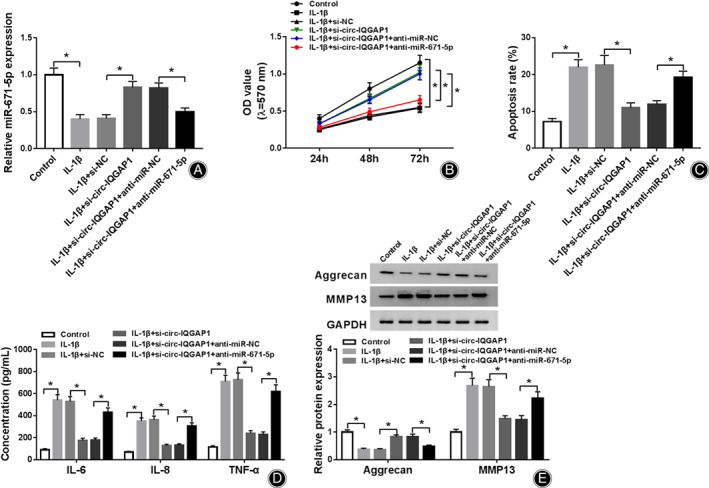
The influence of circ‐IQGAP1 and miR‐671‐5p on IL‐1β‐caused cell apoptosis, inflammatory injury, and extracellular matrix degradation. miR‐671‐5p expression (A), cell viability (B), apoptosis (C), IL‐6, IL‐8 and TNF‐α levels (D) and aggrecan and MMP13 abundances (E) were measured in CHON‐001 cells with transfection of si‐NC, si‐circ‐IQGAP1, si‐circ‐IQGAP1 + anti‐miR‐NC or anti‐miR‐671‐5p after treatment of IL‐1β. ^*^ *P* < 0.05.

To test whether miR‐671‐5p was required for circ‐IQGAP1 in regulating IL‐1β‐induced chondrocyte injury, CHON‐001 cells were transfected with si‐NC, si‐circ‐IQGAP1, si‐circ‐IQGAP1 + anti‐miR‐NC or anti‐miR‐671‐5p, and then treated *via* 10 ng/mL of IL‐1β. miR‐671‐5p expression was evidently elevated *via* circ‐IQGAP1 silence, which was weakened *via* miR‐671‐5p knockdown (Fig. 4A). Moreover, miR‐671‐5p knockdown abated the effect of circ‐IQGAP1 silence on cell viability (Fig. 4B). Additionally, miR‐671‐5p down‐regulation mitigated the suppressive function of circ‐IQGAP1 knockdown on IL‐1β‐caused apoptosis (Fig. 4C). Furthermore, miR‐671‐5p knockdown alleviated silence of circ‐IQGAP1‐mediated inhibitive role in the secretion of IL‐6, IL‐8, and TNF‐α (Fig. 4D). Besides, miR‐671‐5p down‐regulation reversed the influence of circ‐IQGAP1 interference on expression of aggrecan and MMP13 (Fig. 4E). These results indicated that circ‐IQGAP1 regulated IL‐1β‐induced chondrocyte injury *via* miR‐671‐5p.

### TCF4 is Targeted *via* miR‐671‐5p

**Fig. 5:**
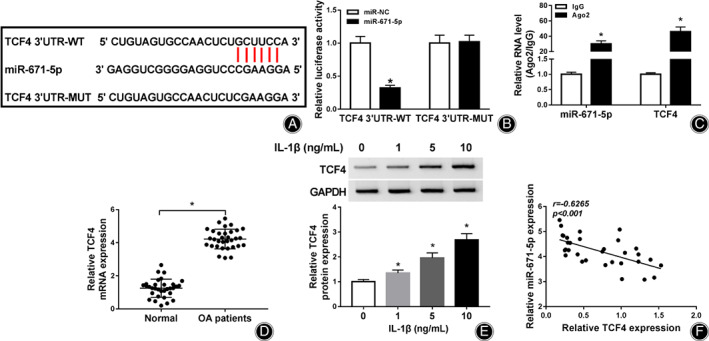
The target correlation of miR‐671‐5p and TCF4. (A) The target sequence of miR‐671‐5p and TCF4. (B) Luciferase activity was examined in CHON‐001 cells with transfection of TCF4 3′UTR‐WT or TCF4 3′UTR‐WT and miR‐671‐5p mimic or miR‐NC. (C) miR‐671‐5p and TCF4 abundances after RIP. (D) TCF4 abundance in the cartilage tissues of OA or normal patients. *n* = 32. (E) TCF4 expression in CHON‐001 cells after treatment of various doses of IL‐1β for 24 h. (F) The linear correlation of miR‐671‐5p and TCF4 in OA patients. ^*^ *P* < 0.05.

To further analyze the regulatory network addressed *via* circ‐IQGAP1/miR‐671‐5p, the targets of miR‐671‐5p were predicted *via* StarBase. TCF4 was predicted as a target of miR‐671‐5p, and the target sequence was displayed in Fig. 5A. To identify the interaction between miR‐671‐5p and TCF4, we performed the dual‐luciferase reporter analysis after the construct of TCF4 3′UTR‐WT and TCF4 3′UTR‐MUT. As exhibited in Fig. 5B, miR‐671‐5p addition evidently reduced the luciferase activity of TCF4 3′UTR‐WT, but did not change the luciferase reporter activity of TCF4 3′UTR‐MUT (Fig. 5B). Furthermore, there were amounts of miR‐671‐5p and TCF4 enriched in the same complex *via* Ago2 RIP (Fig. 5C). In addition, TCF4 expression was evidently enhanced in OA patients and CHON‐001 cells challenged *via* IL‐1β (Figs 5D and E). Besides, TCF4 expression in OA patients was inversely associated with miR‐671‐5p (*r* = −0.6265, *P* < 0.001) (Fig. 5F). These results suggested that TCF4 was targeted *via* miR‐671‐5p.

### miR‐671‐5p Overexpression Attenuates IL‐1β‐Caused Cell Apoptosis, Inflammatory Injury, and Extracellular Matrix Degradation *via* Regulating TCF4

**Fig. 6:**
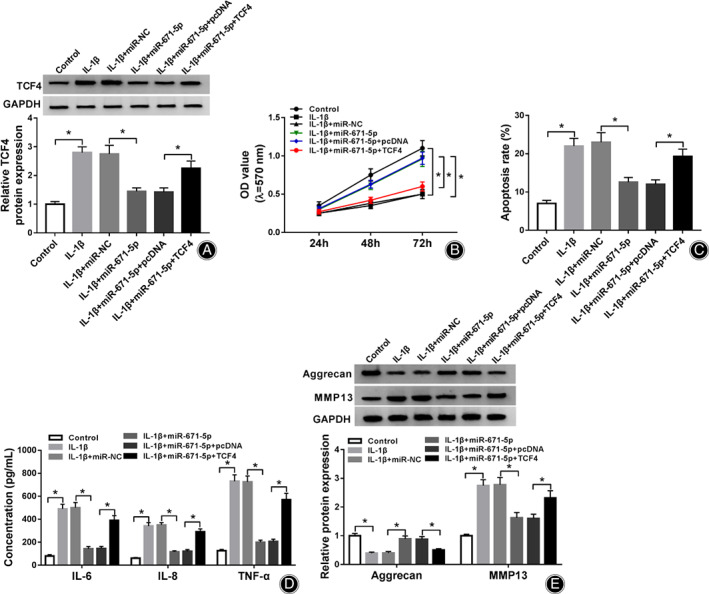
The influence of miR‐671‐5p and TCF4 on IL‐1β‐caused cell apoptosis, inflammatory injury, and extracellular matrix degradation. TCF4 level (A), cell viability (B), apoptosis (C), IL‐6, IL‐8 and TNF‐α levels (D) and aggrecan and MMP13 abundances (E) were examined in CHON‐001 cells with transfection of miR‐NC, miR‐671‐5p mimic, miR‐671‐5p mimic + pcDNA or TCF4 overexpression vector after treatment of IL‐1β. ^*^ *P* < 0.05.

To analyze the function of miR‐671‐5p in IL‐1β‐caused chondrocyte damage and explore whether it required TCF4, CHON‐001 cells were transfected with miR‐NC, miR‐671‐5p mimic, miR‐671‐5p mimic + pcDNA or TCF4 overexpression vector before exposure to 10 ng/mL of IL‐1β. TCF4 abundance was markedly declined *via* miR‐671‐5p addition, which was up‐regulated *via* transfection of TCF4 overexpression vector (Fig. 6A). Furthermore, miR‐671‐5p overexpression increased the viability of IL‐1β‐challenged CHON‐001 cells, which was abated *via* TCF4 up‐regulation (Fig. 6B). In addition, miR‐671‐5p overexpression mitigated IL‐1β‐caused apoptosis, and this effect was abolished *via* TCF4 restoration (Fig. 6C). Moreover, miR‐671‐5p overexpression attenuated IL‐1β stimulated the secretion of IL‐6, IL‐8, and TNF‐α, and this effect was reversed *via* TCF4 up‐regulation (Fig. 6D). Besides, miR‐671‐5p overexpression alleviated IL‐1β‐caused extracellular matrix degradation *via* regulating aggrecan and MMP13, and this event was abated *via* TCF4 overexpression (Fig. 6E). These data indicated that miR‐671‐5p regulated IL‐1β‐induced chondrocyte injury *via* TCF4.

### circ‐IQGAP1 Silence Decreases TCF4 Expression *via* miR‐671‐5p

**Fig. 7:**
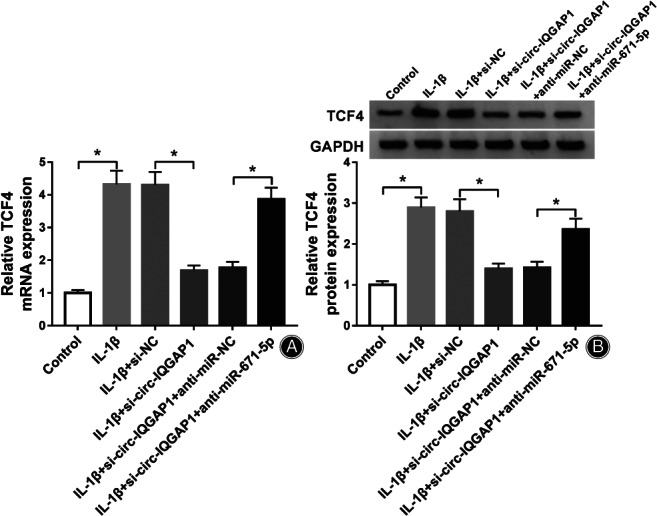
The influence of circ‐IQGAP1 and miR‐671‐5p on TCF4 expression. (A and B) TCF4 expression in CHON‐001 cells with transfection of si‐NC, si‐circ‐IQGAP1, si‐circ‐IQGAP1 + anti‐miR‐NC or anti‐miR‐671‐5p after treatment of IL‐1β. ^*^ *P* < 0.05.

**Fig. 8:**
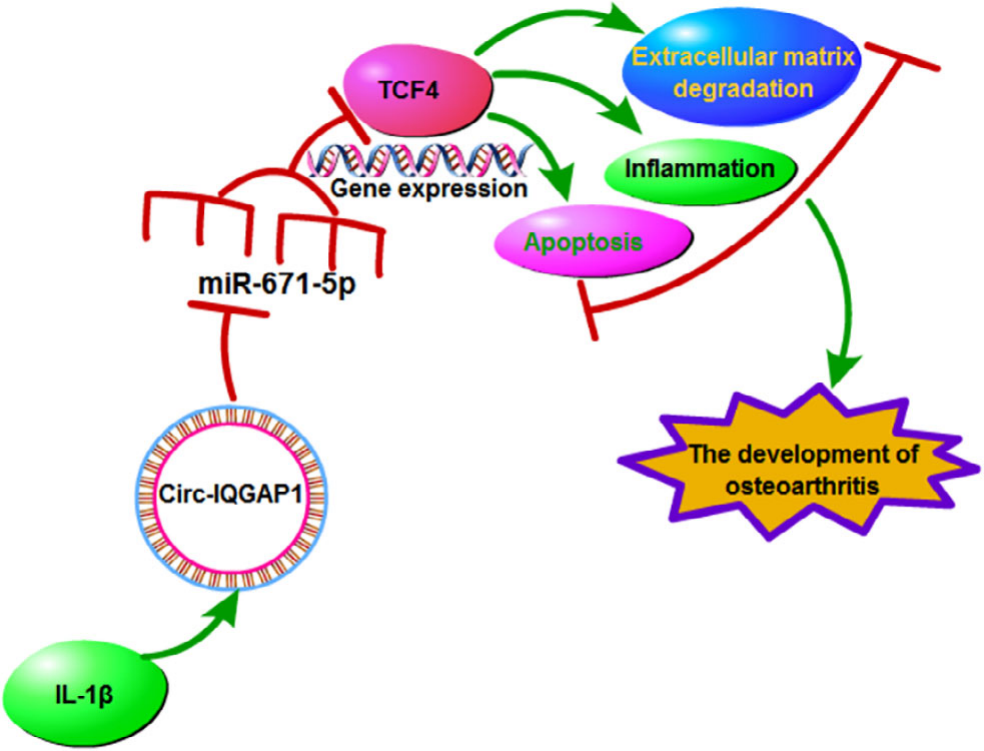
The schematic diagram of the involvement of the circ‐IQGAP1/miR‐671‐5p/TCF4 axis in OA development. In IL‐1β‐treated chondrocytes, circ‐IQGAP1 expression was enhanced. Increased circ‐IQGAP1 sponges miR‐671‐5p to upregulate TCF4 expression, thus to promote IL‐1β‐caused chondrocyte apoptosis, inflammatory injury, and extracellular matrix degradation during OA.

To determine how and whether circ‐IQGAP1 regulated TCF4, CHON‐001 cells were transfected with si‐NC, si‐circ‐IQGAP1, si‐circ‐IQGAP1 + anti‐miR‐NC or anti‐miR‐671‐5p, and then exposed to IL‐1β. As shown in Fig. 7A,B, TCF4 expression was evidently up‐regulated in CHON‐001 cells after exposure to IL‐1β. Besides, TCF4 abundance was remarkably reduced *via* circ‐IQGAP1 silence, which was restored *via* miR‐671‐5p knockdown. These results suggested that circ‐IQGAP1 could regulate TCF4 *via* binding to miR‐671‐5p. Then, the circ‐IQGAP1/miR‐671‐5p/TCF4 axis activated by IL‐1β could promote chondrocyte apoptosis, inflammatory injury, and extracellular matrix degradation, which contributed to OA development (Fig. 8).

## Discussion

OA is a frequently age‐associated disorder in adults accompanied with severe pain^4^, ^33^. The OA pathogenesis is relevant to noncoding RNAs, such as circRNAs and miRNAs^34^. circRNAs have vital roles in regulating cartilage degeneration in OA^12^. In this research, we established the cellular model of OA using IL‐1β‐challenged CHON‐001 cells and explored the function and mechanism of circ‐IQGAP1 in IL‐1β‐caused chondrocyte injury. This study was the first to confirm that circ‐IQGAP1 knockdown could mitigate IL‐1β‐induced injury, and this was mediated *via* miR‐671‐5p/TCF4 axis.

### Function of circ‐IQGAP1 in OA

A previous study showed that circ‐IQGAP1 abundance was elevated in synovial fluid from OA patients, and might have important clinical value *via* acting as a diagnostic biomarker for OA^18^. Similarly, here we also found the increased circ‐IQGAP1 in OA cartilage samples and IL‐1β‐challenged chondrocytes, implying that circ‐IQGAP1 might be involved in IL‐1β‐caused chondrocyte damage. The chondrocyte apoptosis is the contributor of cartilage degeneration in OA^6^. Through detecting the cell viability and apoptotic rate under IL‐1β, we found that circ‐IQGAP1 knockdown mitigated IL‐1β‐caused chondrocyte apoptosis. The inflammation is also an important factor in degenerative joint disease like OA^35^. By detecting the secretion of pro‐inflammatory cytokines (IL‐6, IL‐8 and TNF‐α), our study confirmed that circ‐IQGAP1 silence attenuated IL‐1β‐induced inflammatory injury. The destruction of extracellular matrix in articular cartilage is another key factor in OA progression^8^. MMP13 is a key enzyme responsible for the degenerative process in cartilage^36^. The inhibition of anabolic factor aggrecan and promotion of catabolic factor MMP13 indicate the degradation of extracellular matrix in cartilage tissues, which contributes to the cartilage injury in OA^37^. In this research, we found that extracellular matrix degradation was indeed caused *via* IL‐1β, revealed *via* the reduced aggrecan and increased MMP13. Moreover, circ‐IQGAP1 silence weakened this injury. Collectively, circ‐IQGAP1 knockdown protected against IL‐1β‐caused chondrocyte damage, indicating circ‐IQGAP1 might function as a therapy target for regulation of chondrocyte damage in OA.

### Target Association of circ‐IQGAP1 and miR‐671‐5p in OA

The circRNAs are discovered as miRNA sponges to participate in the progression of human diseases, including OA^38^. Previous study indicted miR‐671‐5p level was declined in plasma and cartilage samples of OA patients, and miR‐671‐5p could mitigate IL‐1β‐induced chondrocyte damage and OA progression in mice^21^. Similarly, we also found the lowly expressed miR‐671‐5p in IL‐1β‐challenged CHON‐001 cells. Moreover, miR‐671‐5p overexpression attenuated IL‐1β‐caused chondrocyte apoptosis, inflammatory injury, and extracellular matrix degradation, indicating the protective function of miR‐671‐5p in IL‐1β‐induced OA‐like damage. Besides, we validated that miR‐671‐5p was targeted *via* circ‐IQGAP1, and miR‐671‐5p knockdown abolished the influence of circ‐IQGAP1 silence on chondrocyte injury, suggesting that circ‐IQGAP1 might regulate IL‐1β‐induced OA progression *in vitro via* sponging miR‐671‐5p.

### The Importance of circ‐IQGAP1/miR‐671‐5p/TCF4 Axis in OA

Next, we explored the target of miR‐671‐5p and identified that TCF4 was targeted *via* miR‐671‐5p. Ma *et al*. reported that TCF4 was a pro‐catabolic and apoptotic factor *via* promoting the nuclear factor κB (NF‐κB) pathway^39^. Moreover, TCF4 contributed to lipopolysaccharide‐induced OA progression *via* facilitating chondrocyte damage^40^. Additionally, TCF4 promoted IL‐1β‐caused chondrocyte apoptosis and extracellular matrix degradation^24^. These reports have proposed the promoting role of TCF4 in IL‐1β‐caused OA‐like damage. In this research, we also found the poor function of TCF4 *via* rescue experiments, in which TCF4 overexpression abrogated the influence of miR‐671‐5p on chondrocyte injury. Besides, our study displayed that circ‐IQGAP1 could modulate TCF4 level *via* binding miR‐671‐5p in chondrocytes under IL‐1β. Thus, we concluded that circ‐IQGAP1 could target TCF4 *via* miR‐671‐5p to participate in OA progression.

### Conclusion

In conclusion, circ‐IQGAP1 knockdown alleviated IL‐1β‐induced chondrocyte damage, possibly *via* regulating the miR‐671‐5p/TCF4 axis. Our study proposes a new insight into the pathogenesis of OA‐like chondrocyte damage and indicates that circ‐IQGAP1 might act as a novel target for OA treatment.
